# Intelligent Control of Magnetic Ball Suspension Systems via a Novel Hyperbolic Tangent PID Controller Tuned by the Artificial Lemming Algorithm

**DOI:** 10.3390/biomimetics11030205

**Published:** 2026-03-11

**Authors:** Serdar Ekinci, Davut Izci, Vedat Tümen, Mostafa Jabari, Emre Çelik, Ali Elrashidi

**Affiliations:** 1 Department of Computer Engineering, Bitlis Eren University, 13100 Bitlis, Turkey; sekinci@beu.edu.tr; 2 Department of Electrical and Electronics Engineering, Bursa Uludag University, 16059 Bursa, Turkey; davutizci@uludag.edu.tr; 3 Faculty of Electrical Engineering, Sahand University of Technology, Tabriz 51335, Iran; m_jabari97@sut.ac.ir; 4 Department of Electrical and Electronics Engineering, Düzce University, 81600 Düzce, Turkey; emrecelik@duzce.edu.tr; 5 Department of Electrical Engineering, University of Business and Technology, Jeddah 23435, Saudi Arabia

**Keywords:** adaptive cost function, artificial lemming algorithm, hyperbolic tangent-based controller, magnetic ball suspension system, optimization

## Abstract

Magnetic ball suspension (MBS) systems are widely used as benchmark platforms in control engineering due to their nonlinear dynamics and inherent open-loop instability, which pose substantial challenges for conventional linear control strategies. The objective of this study is to investigate a hyperbolic tangent–based proportional–integral–derivative (tanh-PID) control structure for MBS systems and to assess the suitability of the artificial lemming algorithm (ALA) for tuning its parameters within a simulation-based benchmark framework. The proposed approach embeds smooth nonlinear signal shaping through the hyperbolic tangent function directly into the classical PID structure, while controller parameters are obtained via metaheuristic optimization using ALA. A performance index balancing overshoot suppression and tracking error minimization is adopted, and the controller is evaluated on a linearized MBS model to ensure comparability with existing studies. Simulation results demonstrate that the optimized tanh-PID controller achieves improved transient and steady-state performance, including a rise time of 0.0144 s, settling time of 0.0275 s, overshoot of 2.98%, and a steady-state error of 2.69 × 10^−5^, when compared with classical PID, fractional-order PID (FOPID), and real PID with second-order derivative (RPIDD^2^) controllers under identical conditions. The results indicate that bounded nonlinear preprocessing combined with metaheuristic-based parameter tuning can provide an effective and practical control alternative for unstable nonlinear systems such as magnetic ball suspension systems.

## 1. Introduction

Magnetic ball suspension (MBS) systems are well-known benchmark platforms in control engineering due to their highly nonlinear dynamics, third-order system characteristics, and inherent open-loop instability [1,2,3,4,5]. These systems naturally diverge from equilibrium without continuous corrective input, making stabilization and precise control particularly difficult [6]. As a result, they have become a standard benchmark for testing advanced control algorithms [7]. In this paper, a hyperbolic tangent–based PID (tanh-PID) control structure is investigated for magnetic ball suspension systems, with controller parameters obtained through optimization using the artificial lemming algorithm (ALA) [8]. To the authors’ knowledge, this represents the first application of ALA to the tuning of a tanh-shaped PID controller for an MBS benchmark system. This innovative control scheme integrates a smooth nonlinear preprocessing stage with an optimization mechanism to improve robustness, noise immunity, and transient performance. By employing an adaptive cost function that balances overshoot suppression and error minimization, the proposed controller achieves superior performance compared to classical approaches.

Conventional PID controllers remain the most widely adopted approach in industrial control due to their simplicity, low implementation cost, and satisfactory performance in linear, well-modeled systems [9,10,11,12]. However, in complex and nonlinear systems like MBS, conventional PID controllers may struggle to deliver optimal performance [13,14,15]. To address these limitations, advanced controllers such as fractional-order PID (FOPID) [16,17,18,19] and real PID with second-order derivative (RPIDD^2^) [20] have been developed, offering greater flexibility but posing challenges in parameter tuning. Metaheuristic optimization algorithms, such as the ALA [8], starfish optimization algorithm (SFOA) [21], liver cancer algorithm (LCA) [22], and improved artificial electric field algorithm (iAEFA) [23], have emerged as powerful tools for tuning controller parameters [24,25,26,27,28]. These algorithms, inspired by natural behaviors, efficiently explore the search space to find optimal parameter sets.

Recent studies have introduced a variety of advanced control techniques to enhance the performance of MBS systems. Ekinci et al. [29] presented a hybrid whale optimization algorithm with simulated annealing (WOA-SA) to fine-tune PID parameters, resulting in improved stability and transient behavior. Demirören et al. [30] proposed an opposition-based artificial electric field (ObAEF) algorithm to optimize a fractional-order PID (FOPID) controller, significantly reducing overshoot and settling time. Wei et al. [31] employed an improved Adagrad algorithm with a deep neural network feedforward compensator, achieving high tracking accuracy without explicit modeling. Verma and Padhy [32] introduced a novel integral tan hyperbolic square error (ITHSE) performance index for PID tuning via genetic algorithm (GA), enhancing robustness and reducing control effort compared to classical ISE. Additional efforts by Ekinci, et al. [33] explored advanced structures like RPIDD^2^ controllers and hybrid optimization frameworks, further validating the potential of enhanced metaheuristics in handling MBS nonlinearities. In a more recent study, Tang et al. [34] introduced a fuzzy inference-based neural network compensation controller that adaptively adjusts control output to address poor transient performance of undertrained networks, significantly improving overshoot and settling time. Furthermore, Jastrzębski and Kabziński [35] proposed a fuzzy model inversion-based adaptive controller capable of accurate trajectory tracking despite unknown system parameters, with experimental validation on a real DSP-controlled maglev system.

Furthermore, several very recent studies have further diversified the control landscape for MBS systems. Jastrzębski and Kabziński [35] introduced an adaptive controller based on fuzzy model inversion, which, through Lyapunov stability analysis and DSP hardware implementation, demonstrated accurate tracking capability and robustness against parameter variations. Pedroso et al. [36] proposed a hyperbolic tangent adaptive PID + LQR control applied to a step-down converter, in which the tanh function was used within an adaptive framework to enhance robustness, but without direct integration with a metaheuristic optimization algorithm. Similarly, Yavuz and İkizoğlu [37] employed a hyperbolic tangent adaptive LQR + PID controller for a quadrotor, leveraging the tanh nonlinearity to improve stability, but their approach relied on LQR-based gain computation rather than real-time adaptive tuning via nature-inspired optimization. These studies confirm that hyperbolic tangent nonlinearities have previously been incorporated within PID-related control frameworks, typically in combination with LQR-based designs. In contrast, the present work examines a standalone tanh-shaped PID structure whose parameters are optimized using a metaheuristic algorithm. While neither the tanh nonlinearity nor the optimization paradigm is new in itself, the specific controller formulation and its optimization using the artificial lemming algorithm for the magnetic ball suspension problem have not been reported previously.

Qiang et al. [38] combined nonlinear NARX modeling with a backstepping design and parameter estimation via RLS-FF, improving dynamic performance and disturbance rejection capability. Tang et al. [34] developed a hybrid structure consisting of fuzzy inference, a recurrent neural network, and a basic PID controller, significantly reducing overshoot and settling time. In the field of predictive control, Peng et al. [39] proposed a deep learning-based model predictive controller for the magnetic levitation ball system, enhancing accuracy and responsiveness by forecasting dynamic behavior. Moreover, an improved linear active disturbance rejection control (I-LADRC) method introduced for magnetic suspension systems provided greater stability and robustness against environmental disturbances [40].

Despite the significant progress reported in the literature, several limitations remain evident. First, many existing PID, FOPID, and RPIDD^2^ designs rely primarily on linear error processing, which may lead to excessive transient control effort and overshoot in inherently unstable MBS dynamics. Second, although advanced learning-based and adaptive controllers have demonstrated promising performance, they often introduce considerable structural complexity, training requirements, and computational overhead that may hinder practical deployment in resource-constrained environments. Third, while numerous metaheuristic optimizers have been applied to controller tuning, the artificial lemming algorithm has not yet been explored in the context of hyperbolic tangent–shaped PID control for magnetic ball suspension systems.

These observations motivate the present study, which aims to develop a structurally simple yet performance-enhanced control framework by integrating bounded nonlinear signal shaping with an effective metaheuristic tuning strategy. Consequently, the present study examines a hyperbolic tangent–based PID controller in which smooth nonlinear signal shaping is embedded directly within the proportional, integral, and derivative channels. The controller parameters are obtained through optimization using the artificial lemming algorithm. Unlike strictly adaptive control schemes in the Lyapunov sense, the tuning process in this work is supervisory and simulation-based, yielding fixed controller parameters that are subsequently evaluated under identical test conditions. The hyperbolic tangent function is employed to bound and smoothly scale the control signals, which can mitigate excessive control action and improve transient smoothness in unstable operating regimes.

The selection of the hyperbolic tangent nonlinearity is motivated by the need for smooth and bounded signal shaping in the magnetic ball suspension system, which is inherently nonlinear and open-loop unstable. Unlike conventional linear PID action, the tanh function introduces a continuous soft-limiting effect that suppresses excessive control effort and reduces overshoot without introducing discontinuities or chattering. This property is particularly beneficial for magnetic levitation dynamics, where abrupt control variations may degrade stability.

The artificial lemming algorithm is employed as the parameter tuning mechanism due to its adaptive exploration–exploitation balance and its reported effectiveness in complex nonlinear optimization problems. The tanh-PID structure involves an eight-dimensional parameter space with strong coupling between gains and shaping coefficients, making manual tuning or classical methods inefficient. ALA provides a robust supervisory optimization framework capable of reliably identifying high-quality parameter sets under identical benchmarking conditions. Together, the bounded nonlinear preprocessing and ALA-based tuning form a coherent control design aimed at improving transient smoothness, robustness, and overall regulation performance for the MBS benchmark.

The main contributions of this paper can be summarized as follows:

• Investigation of a tanh-shaped PID control structure for magnetic ball suspension systems, focusing on bounded nonlinear signal shaping within a classical PID framework.
• First reported application of the ALA to the tuning of a tanh-shaped PID controller and to the magnetic ball suspension benchmark problem.
• Systematic simulation-based benchmarking against PID, FOPID, and RPIDD^2^ controllers, as well as against several established metaheuristic tuning strategies, under identical modeling and evaluation conditions.
• Reproducible performance analysis highlighting the effects of nonlinear preprocessing and optimizer choice on transient and steady-state behavior.

The remainder of the paper is organized as follows. Section 2 briefly outlines the artificial lemming algorithm and its optimization principles. Section 3 presents the mathematical model and linearization of the magnetic ball suspension system. Section 4 introduces the tanh-PID control structure. Section 5 describes the optimization framework and performance index. Section 6 reports and discusses the comparative simulation results. Finally, Section 7 concludes the paper and outlines directions for future research.

## 2. Artificial Lemming Algorithm

**Figure 1:**
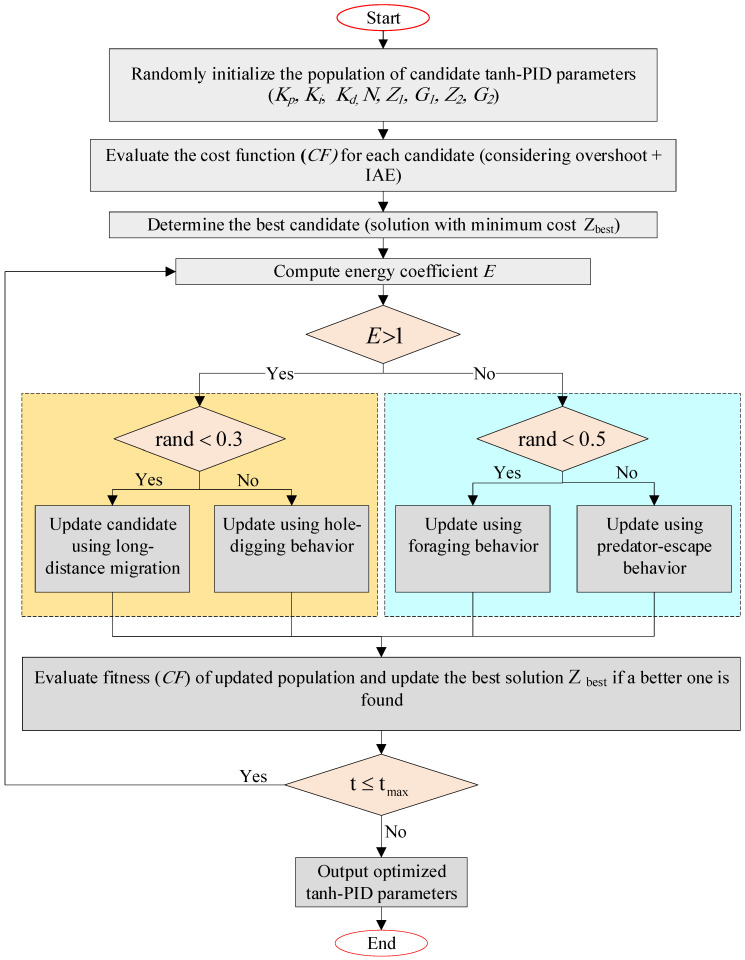
Working mechanism of ALA.

The artificial lemming algorithm (ALA) is a novel nature-inspired meta-heuristic optimization algorithm that mimics the real-world survival behaviors of lemmings [41]. It was developed to address the common shortcomings of traditional meta-heuristics, such as premature convergence and limited adaptability in high-dimensional or complex search spaces. Inspired by the behavioral patterns of lemmings in their natural habitat, ALA introduces four biologically meaningful strategies: long-distance migration, digging holes, foraging, and evading predators. These strategies enable a robust and adaptive balance between exploration (searching new areas) and exploitation (refining promising solutions), which is critical for solving nonlinear, constrained, and real-world optimization problems [8]. The working mechanism of artificial lemming algorithm is shown in Figure 1. Lemmings are small rodents native to the Arctic tundra, known for their group behavior, migration, and survival instincts. ALA models the following behaviors. These behaviors are embedded into mathematical models, each contributing to different phases of the optimization process [42,43,44].

• **Long-distance migration**: When resources are scarce, lemmings migrate to new areas. In ALA, this behavior supports broad exploration using Brownian motion and direction-switching.
• **Digging holes**: Lemmings dig burrows to escape predators and store food. In ALA, this contributes to moderate local exploration near promising areas.
• **Foraging**: Lemmings search for food within their territory. This is modeled using a spiral-based local search mechanism to intensify exploitation.
• **Evading predators**: Lemmings detect threats and flee rapidly. ALA simulates this through Lévy flight-based rapid movement to escape local optima.

### 2.1. Algorithmic Structure

ALA begins by initializing a population of candidate solutions within the search bounds. Each “lemming” (i.e., candidate solution) updates its position using one of the four behavior models, depending on a dynamic energy factor E(t), which decreases over time. This factor governs the transition from exploration-dominant strategies (migration, digging) to exploitation-dominant strategies (foraging, evasion). Exploration (when E(t)>1):

• **Long-distance migration:**

(1) $${\vec{z}}_{i}\left(t+1\right)={\vec{z}}_{best}\left(t\right)+F\times \vec{BM}\times \left(\vec{R}\times \left({\vec{z}}_{best}\left(t\right)-{\vec{z}}_{i}\left(t\right)\right)+(1-\vec{R})\times ({\vec{z}}_{i}\left(t\right)-{\vec{z}}_{a}\left(t\right))\right)$$

where BM represents Brownian motion, F is a directional flag, and $\vec{R}$ is a random vector.

• **Digging holes:**

(2) $${\vec{z}}_{i}\left(t+1\right)={\vec{z}}_{i}\left(t\right)+F\times L\times \left({\vec{z}}_{best}\left(t\right)-{\vec{z}}_{b}\left(t\right)\right)$$

where L is a dynamic digging factor based on the iteration number. Exploitation (when E(t)≤1):

• **Foraging:**

(3) $${\vec{z}}_{i}\left(t+1\right)={\vec{z}}_{best}\left(t\right)+F\times G\times Levy(d)\times \left({\vec{z}}_{best}\left(t\right)-{\vec{z}}_{i}\left(t\right)\right)$$

with $G=2\times \left(1-\frac{t}{T_{max}}\right)$, allowing the escape intensity to fade over time.

In Equations (1)–(3), $Z_{i}(t)$ denotes the position vector of the i-th lemming (candidate solution) at iteration t, while $Z_{best}(t)$ represents the current global best solution in the population. The term $Z_{a}(t)$ denotes a randomly selected lemming position, and $Z_{b}(t)$ refers to a neighboring or reference lemming used during the digging phase. BM represents Brownian motion, Levy(d) denotes the Lévy flight distribution in d-dimensional space, and F is a directional control flag. The vector $\overset{ˉ}{R}$ is a uniformly distributed random vector in [0,1]. The parameter L denotes the dynamic digging coefficient, and G is the time-varying escape factor defined in Equation (3).

### 2.2. Energy-Driven Transition Mechanism

ALA features a unique energy-decreasing mechanism given in Equation (4). This probabilistically ensures that early iterations favor exploration and later iterations promote exploitation. The transition probability $P\left\{E(t)>1\right\}\approx 0.5$, ensuring a smooth balance between both phases throughout the optimization process.

(4) $$E(t)=4\times arctan\left(1-\frac{t}{T_{max}}\right)\times ln\left(\frac{1}{rand}\right)$$

## 3. Mathematical Modeling of Magnetic Ball Suspension System

**Figure 2:**
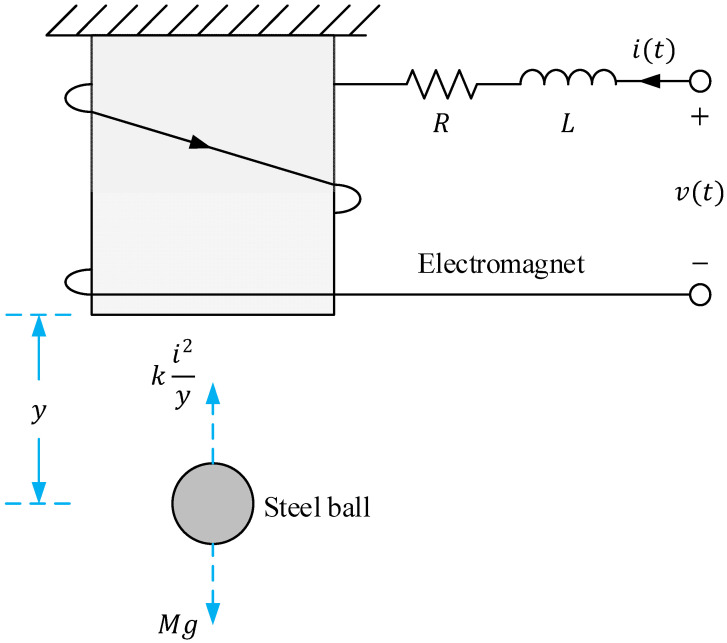
Visual representation of magnetic ball suspension system.

The magnetic ball suspension system is a well-known benchmark in control engineering due to its nonlinear and open-loop unstable nature [45]. It consists of a steel ball suspended vertically beneath an electromagnet. The vertical position of the ball is controlled by modulating the current through the electromagnet, which alters the magnetic force acting on the ball. The system is inherently unstable because, without active control, the ball will either fall due to gravity or be pulled into the magnet. This makes it an ideal testbed for advanced controller design and evaluation [29,30]. A visual representation of the system setup is shown in Figure 2, while the physical and electrical parameters of the system used in this study are provided in Table 1, based on reference [46].

### 3.1. Linearization and Transfer Function Derivation

The magnetic force $F_{m}$ generated by the coil is nonlinear with respect to the current i and the ball position y. Around the equilibrium point $y_{0}$, the system is linearized to facilitate control system design. The resulting linear model captures the core dynamics of the system while enabling the use of classical control techniques. From the linearized equations, the transfer function of the plant from the control voltage (input) to the ball position (output) is obtained as a third-order system [29,30].

(5) $$G\left(s\right)=\frac{a}{\left(s-s_{1})(s-s_{2})(s-s_{3}\right)}$$

where $a=-\frac{2}{L}\sqrt{\frac{kg}{My_{0}}}=-885.8894$, $s_{1}=-\frac{R}{L}=-100$, $s_{2}=\sqrt{g/y_{0}}=4.4294$ and $s_{3}=-\sqrt{\frac{g}{y_{0}}}=-4.4294$, respectively. Substituting the above into the expression, we obtain the transfer function:

(6) $$G\left(s\right)=\frac{-885.9}{\left(s+100)(s-4.4294)(s+4.4294\right)}=\frac{-885.9}{s^{3}+100s^{2}-19.62s-1962}$$

This transfer function reveals the system’s structure: it has one fast stable pole at s=−100, one slow stable pole at s=−4.4294, and one unstable pole at s=4.4294. The presence of the right-half-plane (RHP) pole confirms that the system is open-loop unstable, meaning any deviation from equilibrium will grow over time unless active feedback is applied.

The linearized MBS model employed in this study represents system behavior in the vicinity of the nominal operating point and is widely used in the literature for benchmark-oriented controller evaluation. This choice enables direct and fair comparison with previously reported PID-, FOPID-, and RPIDD^2^-based methods under identical conditions. It is emphasized, however, that linearization does not capture large-signal nonlinear dynamics, actuator saturation, or operating-point shifts. Consequently, the results presented in this paper demonstrate closed-loop performance and stabilization capability for the standard benchmark model and should not be interpreted as global guarantees for the full nonlinear plant. Investigation of the proposed controller under the complete nonlinear dynamics and extended operating regimes constitutes a natural and important direction for future work.

### 3.2. Open-Loop Dynamics

**Figure 3:**
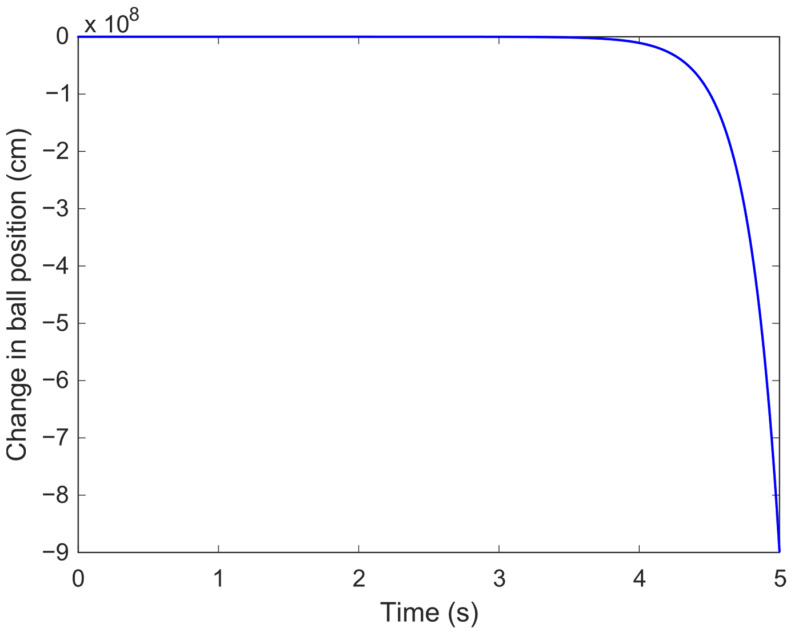
Open-loop step response.

The open-loop step response of the system, shown in Figure 3, illustrates the practical implication of this instability. Upon the application of a unit step input, the ball’s position diverges rapidly instead of converging to a new steady state. This is directly attributable to the system’s unstable pole, which dominates the response over time. Without control, the system cannot maintain the ball at a desired height, as it lacks any natural restoring force. Thus, a controller is required not only to stabilize the system but also to deliver fast, accurate, and smooth positioning.

In Figure 3, a unit step voltage is applied to the electromagnet at t=0 and then held constant throughout the simulation; the input is not removed or set to zero at any later time. The observed divergence arises from the intrinsic open-loop instability of the linearized MBS model, which contains a right-half-plane pole. Under open-loop conditions, a constant voltage does not provide the precise equilibrium force required to counterbalance gravity, and even small deviations from the operating point grow exponentially. Consequently, the ball does not “stick” to a fixed position but instead departs from equilibrium, highlighting the necessity of closed-loop feedback control for stable levitation.

### 3.3. Motivation for Advanced Control

Due to the nonlinear force–position relationship, third-order dynamics, and open-loop instability, the magnetic ball suspension system poses significant challenges to conventional linear control strategies. These challenges justify the adoption of advanced nonlinear or adaptive control methods, such as the tanh-PID controller proposed in this study, which offer better robustness, noise rejection, and nonlinear compensation than standard PID controllers. In the following sections, we develop, tune, and validate a novel tanh-PID control structure capable of effectively stabilizing the system and delivering high-performance tracking across a range of dynamic conditions. It is worth noting that, despite the use of a linearized plant model for benchmarking purposes, the proposed tanh-PID controller itself introduces a bounded nonlinear mapping through the hyperbolic tangent function. Therefore, the closed-loop system is inherently nonlinear around the operating region, and the reported improvements reflect the controller’s capability to handle nonlinear signal characteristics within the standard MBS evaluation framework.

## 4. Proposed Novel Hyperbolic Tangent PID (Tanh-PID) Controller

**Figure 4:**
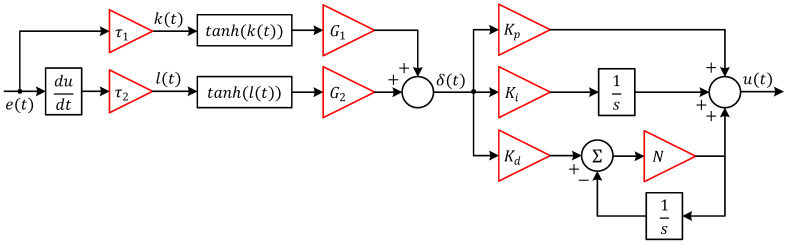
Block diagram of proposed novel tanh-PID controller.

This section presents the design and formulation of a novel nonlinear controller referred to as the tanh-PID controller, which augments the conventional PID architecture by introducing a hyperbolic tangent-based nonlinear preprocessing of the error signal and its derivative. The objective is to enhance the controller’s performance in terms of robustness, smoothness, and noise immunity, particularly in nonlinear and sensitive systems such as the magnetic ball suspension system. The structure of the proposed controller is illustrated in Figure 4.

It consists of two key stages: (1) nonlinear transformation of the error dynamics, and (2) a classical PID controller operating on the preprocessed signal. Let $e\left(t\right)=r\left(t\right)-y(t)$, denote the instantaneous tracking error. The proposed method first transforms the error and its time derivative using hyperbolic tangent nonlinearities. Specifically, the derivative of the error is scaled by a shaping parameter $\tau_{1}$, and passed through a tanh. These two signals are then scaled by output gains $G_{1}$ and $G_{2}$, respectively, and combined to form an intermediate nonlinear signal δ(t), defined as:

(7) $$\delta \left(t\right)=G_{1}\times tanh\left(\;\tau_{1}\times e(t)\right)+G_{2}\times tanh\left(\tau_{2}\times \frac{de(t)}{dt}\right)$$

This signal serves as the input to a conventional PID controller with filtered derivative action. The controller output u(t) is then calculated as:

(8) $$u\left(t\right)=K_{p}\times \delta \left(t\right)+K_{i}\int_{0}^{t}\delta (\tau )d\tau +K_{d}\times \frac{d\delta_{f}(t)}{dt}$$

Here, $K_{P}$, $K_{i}$, and $K_{d}$ are the proportional, integral, and derivative gains, respectively, while N is the filter coefficient used in the first-order low-pass filter applied to the derivative term to attenuate high-frequency noise and $\left(d\delta_{f}(t)/dt\right)=N\times \left(\delta \left(t\right)-\delta_{f}(t)\right)$. This derivative structure can also be expressed in the Laplace domain as:

(9) $$PID\left(s\right)=K_{p}+\frac{K_{i}}{s}+K_{d}\frac{Ns}{s+N}$$

and the overall controller in the Laplace domain becomes:

(10) $$U\left(s\right)=\left(K_{p}+\frac{K_{i}}{s}+K_{d}\frac{Ns}{s+N}\right)\times ∆(s)$$

where ∆(s) is the Laplace transform of $\delta \left(t\right)$. Figure 4 shows that the tanh-based shaping of the error signals acts as a bounded, smooth nonlinearity that suppresses large variations in the derivative or error magnitude, helping to mitigate control signal spikes, reduce overshoot, and enhance robustness. The shaping parameters $\tau_{1}$, $G_{1}$, $\tau_{2}$ and $G_{2}$ provide tuning flexibility to adapt the nonlinear influence on the control behavior, making the controller capable of adjusting its aggressiveness and sensitivity. The nonlinearity introduced in this structure is based on the hyperbolic tangent function, mathematically defined as:

(11) $$\tanh\left(x\right)=\frac{e^{x}-e^{-x}}{e^{x}+e^{-x}}$$

**Figure 5:**
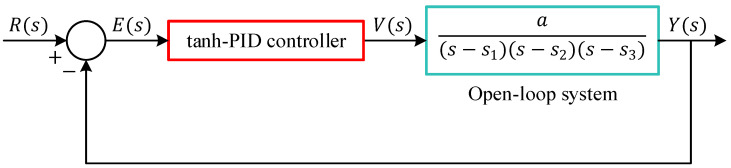
Block diagram of proposed novel tanh-PID controller.

This function is smooth, bounded in the interval (−1, 1), and differentiable, making it ideal for suppressing excessive control effort and handling large variations in the input without causing discontinuities or chattering in the control signal. The complete system structure is further illustrated in Figure 5, where the tanh-PID controller operates in closed-loop with the magnetic suspension plant. This configuration enables the control system to respond effectively to disturbances and nonlinearities while ensuring smooth tracking of reference trajectories.

It is noted that the proposed controller introduces a nonlinear and gain-varying mapping through the hyperbolic tangent function. However, the controller parameters remain fixed during closed-loop operation, and the resulting system is nonlinear but time-invariant. The hyperbolic tangent function is smooth, bounded in the interval (−1, 1), and continuously differentiable. These properties ensure that the preprocessing stage cannot generate unbounded internal signals or discontinuities, and that excessive amplification of large error or derivative values is inherently limited. In this sense, the tanh-based shaping acts as a soft saturation mechanism that mitigates impulsive control action and contributes to well-damped transient behavior. Within the scope of this study, stability is assessed in a practical benchmark sense. The magnetic ball suspension plant exhibits a right-half-plane pole and is open-loop unstable. All reported closed-loop simulations demonstrate bounded trajectories and convergence to the reference for this unstable benchmark under identical conditions. These results confirm closed-loop stabilization for the considered model and operating point. The present work does not claim global or Lyapunov-based stability guarantees for arbitrary nonlinear regimes or parameter variations; rather, it demonstrates consistent stabilization and performance improvement on the standard MBS benchmark, in line with prevailing practice in comparative controller-design studies.

## 5. Implementation of ALA to Magnetic Ball Suspension System

In this section, the proposed ALA-based tanh-PID controller is applied to a magnetic ball suspension system a well-known nonlinear and open-loop unstable system frequently used to benchmark control strategies. The goal is to regulate the position of the steel ball by adjusting the magnetic force through the controller, ensuring stability, minimal overshoot, and precise tracking of a reference position. To guide the optimization of controller parameters, an adaptive cost function (CF) is employed, integrating both transient and steady-state performance aspects. The cost function is defined as:

(12) $$CF=\varphi \times \left(M_{p}-1\right)+\left(1-\varphi \right)\times \int_{0}^{t_{f}}|e\left(t\right)|dt$$

where $M_{p}$, $t_{f}$ are the peak values of y(t) and simulation time, respectively. In addition, $e\left(t\right)=r\left(t\right)-y(t)$ is instantaneous error and weighting coefficient φ=0.12 and $t_{f}=5$ s. This cost function structure enables a trade-off between penalizing excessive overshoot and minimizing the overall error over time. The first term, $\varphi \times \left(M_{p}-1\right)$, emphasizes the control of peak overshoot beyond the desired steady-state value (assumed to be 1). The second term, weighted by 1−φ, focuses on minimizing the integral of absolute error (IAE), which reflects accumulated deviation from the reference over the simulation time.

The search intervals reported in Table 2 were determined based on three complementary considerations. First, the ranges of the PID gains were selected in accordance with commonly reported values in the magnetic ball suspension literature to ensure fair benchmarking and closed-loop stabilizability. Due to the sign convention of the adopted plant model, the proportional, integral, and derivative gains were restricted to negative values, which is consistent with prior MBS control studies. Second, the bounds of the filter coefficient and nonlinear shaping parameters were chosen to maintain physical realizability and to prevent excessive amplification of the preprocessed error signals. In particular, the lower limits ensure sufficient control authority, while the upper limits avoid overly aggressive responses and numerical stiffness during optimization. Third, preliminary sensitivity analyses were conducted to verify that the selected intervals provide a sufficiently wide yet computationally stable search space for the artificial lemming algorithm. These considerations collectively ensure a fair, stable, and practically meaningful optimization framework.

**Figure 6:**
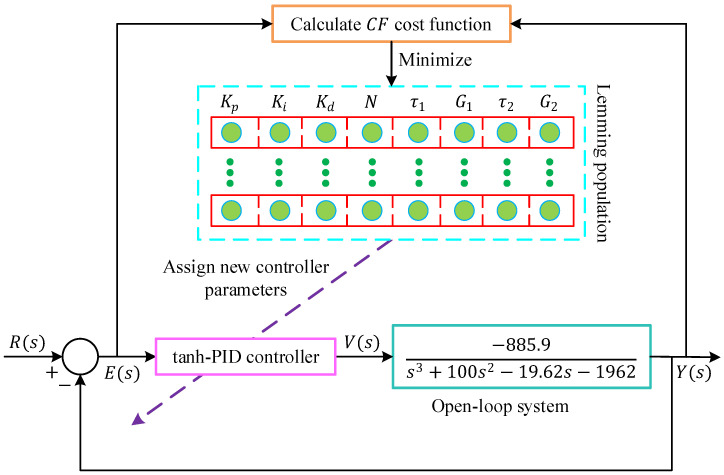
The implementation of the proposed method to the magnetic ball suspension system.

The overall implementation of the proposed control structure is illustrated in Figure 6, which shows the integration of the ALA-based tuning mechanism within the magnetic ball suspension system. In this framework, the ALA is employed as a supervisory, simulation-based optimizer that determines the optimal set of tanh-PID parameters prior to closed-loop operation. The optimization process is carried out offline in the MATLAB/Simulink environment by repeatedly simulating the closed-loop response and minimizing the defined cost function. Once the optimization converges, the obtained controller parameters are fixed and subsequently used during closed-loop execution. No parameter adaptation or learning takes place during real-time operation. Consequently, the proposed scheme does not constitute an adaptive or online tuning controller in the Lyapunov sense; rather, it represents a nonlinear PID structure whose parameters are obtained through offline metaheuristic optimization.

In general, the combination of a well-designed adaptive cost function and the real-time tuning capability of ALA makes the proposed tanh-PID controller a robust and high-performance solution for nonlinear control applications such as magnetic levitation systems. The design of the proposed tanh-PID controller follows a clear rationale: the hyperbolic tangent–based preprocessing stage is introduced to ensure smooth and bounded control action, which prevents abrupt changes that could destabilize the magnetic ball suspension system. This nonlinear mapping enhances robustness against sensor noise and mitigates chattering. The ALA is employed for parameter tuning, enabling the controller to adjust in real time to system parameter variations and external disturbances. Together, these features provide a cohesive design framework that directly addresses the limitations of conventional PID, FOPID, and RPIDD^2^ controllers. All simulations and optimization procedures presented in this study were conducted using the MATLAB/Simulink environment (version 2025b) on a Windows-based personal computer. The computational platform was equipped with a 12th Generation Intel^®^ Core™ i7-1260P processor operating at 2.10 GHz and 16.0 GB of RAM.

## 6. Comparative Simulation Results

### 6.1. Employed Algorithms for Comparison

To ensure a fair and consistent performance evaluation, the proposed ALA-based tanh-PID controller was compared against four well-established metaheuristic algorithms: SFOA, LCA, iAEFA, and L-SHADE. Each algorithm was tasked with optimizing the same set of controller parameters under identical simulation conditions. All algorithms were executed for 25 independent runs to account for stochastic variation. In each run, the maximum number of iterations was set to 50, the population size was fixed at 30, and the optimization problem dimension was eight. The eight decision variables represent the tunable parameters of the tanh-PID controller: proportional gain ${\;K}_{p}$, integral gain $K_{i}$, derivative gain $K_{d}$, filter coefficient N, and four additional shaping parameters $\tau_{1}$, $G_{1}$, $\tau_{2}$ and $G_{2}$ that influence the nonlinear dynamics of the control system.

The specific control parameters used for each algorithm were selected based on their respective original studies and are summarized in Table 3. For the ALA, the switching probability Prob, was set to 0.3 as suggested in [8], which governs the exploration–exploitation mechanism during learning. The SFOA employed an algorithmic gain parameter $G_{P}$, with a value of 0.5 [21], while the LCA used a tumor constant f=1, following the standard setup in [22]. For the iAEFA, two key parameters were configured: the initial Coulomb’s constant $K_{0}$, was set to 500, and the control parameter α, was fixed at 40, in accordance with Ref [23]. Lastly, the L-SHADE algorithm utilized a crossover rate $M_{CR}$, and scaling factor $M_{F}$, both set to 0.5, following [47]. By maintaining uniform simulation settings and appropriate algorithm-specific configurations, the comparison was designed to isolate and evaluate the true optimization capability of each method in tuning the nonlinear tanh-PID controller. The results of this comparative analysis are presented in the following subsections.

It is emphasized that all optimizers are executed under an identical population–iteration budget and control-design protocol. Owing to internal algorithmic mechanisms (e.g., adaptive updates or conditional operations), the exact number of fitness evaluations may differ among methods. Consequently, the comparison is intended to reflect controller-tuning performance under a common iteration-based framework, rather than a strict evolutionary-computation benchmark normalized by equal FE budgets. It should be noted that several recent studies have explored advanced learning-based control strategies for magnetic levitation systems, including deep learning–based predictive control and fuzzy neural network compensation schemes. While these approaches demonstrate promising performance, they belong to a fundamentally different class of data-driven and adaptive control frameworks that typically require dedicated training procedures, higher computational resources, and problem-specific network architectures. In contrast, the focus of the present study is on maintaining a structurally simple and practically deployable PID-type controller whose parameters are obtained via metaheuristic optimization. Therefore, the comparative analysis has been intentionally restricted to controllers of comparable structural complexity (PID, FOPID, and RPIDD^2^ families) to ensure a fair and controlled benchmark. Investigation of hybrid learning-assisted extensions of the proposed tanh-PID controller constitutes an interesting direction for future research.

### 6.2. Distribution and Spread Analysis

**Figure 7:**
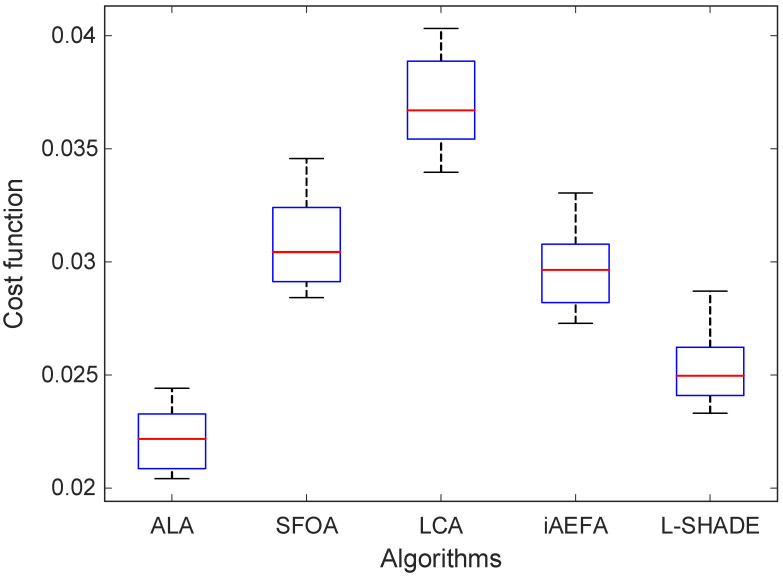
Comparative boxplot analysis.

To further evaluate the consistency and robustness of the optimization methods used for tuning the tanh-PID controller, a distributional analysis was conducted based on the results of multiple independent runs. This analysis provides a visual and statistical representation of the variability in performance for each algorithm. Figure 7 presents the boxplot comparison of the cost function values achieved by the ALA, SFOA, LCA, iAEFA, and L-SHADE algorithms. The boxplots reveal the spread, central tendency, and outliers associated with each optimizer’s output distribution. From this visualization, it is evident that the ALA-based approach not only achieves the lowest median cost value but also exhibits the most compact interquartile range, indicating a highly stable and consistent performance across trials.

In contrast, other methods such as LCA and SFOA show wider spreads and higher median values, suggesting both poorer average performance and greater sensitivity to initial conditions. LCA, in particular, displays a notable number of high-value outliers, reflecting an increased likelihood of suboptimal convergence. Meanwhile, although L-SHADE and iAEFA demonstrate moderately tight distributions, their median performance remains inferior to that of ALA, and their interquartile ranges are comparatively broader. Overall, the distribution analysis supports the conclusion that the ALA-based tanh-PID controller offers not only superior average performance but also greater reliability and robustness. The tight clustering of its results around a low central value confirms the consistency of the ALA in navigating the search space efficiently, avoiding erratic behavior, and converging to optimal or near-optimal solutions across a wide range of runs.

### 6.3. Numerical Evaluation of Statistical Performance

To further validate the robustness and consistency of the proposed ALA-based tanh-PID controller, a statistical analysis was conducted over multiple independent runs of each algorithm. This analysis helps determine the stability and reliability of each optimization method under stochastic initialization and iterative variation. Table 4 summarizes the key statistical metrics obtained for all algorithms, including the minimum, maximum, and average values of the cost function, along with the standard deviation and final performance ranking. These metrics provide insights not only into the best-case performance but also the consistency and reproducibility of each optimization approach.

The ALA-based controller demonstrates the best overall statistical performance. It achieves the lowest minimum cost value (2.0414 × 10^−2^) and the best average cost (2.2148 × 10^−2^), confirming its superior optimization capability across runs. Furthermore, the standard deviation of its results (1.2142 × 10^−3^) is the lowest among all methods, highlighting its remarkable stability and minimal performance variability. This robustness is essential for real-world applications, where consistent behavior across repeated deployments is a critical requirement. By contrast, methods such as LCA and SFOA exhibit significantly higher mean cost values, 3.7037 × 10^−2^ and 3.0730 × 10^−2,^ respectively, as well as greater standard deviations, indicating less reliable convergence and more sensitivity to initial conditions. Although L-SHADE and iAEFA produce relatively better results than LCA and SFOA, their average performances still fall short of the ALA-based controller, and their higher standard deviations suggest greater uncertainty in final outcomes.

Ranking analysis based on overall statistical indicators places the ALA-based tanh-PID controller first, followed by L-SHADE in second place and iAEFA in third. These rankings reaffirm the advantage of integrating an ALA into the control architecture. Its online adaptability not only improves transient and steady-state control but also ensures greater reliability in repeated optimization scenarios. Generally, the statistical evaluation confirms that the ALA-based tanh-PID controller consistently delivers lower cost values with reduced variability, making it not only a high-performing but also a robust and dependable solution for control tasks requiring repeated and consistent performance.

### 6.4. Cost Function Minimization and Optimized Controller Parameters

**Figure 8:**
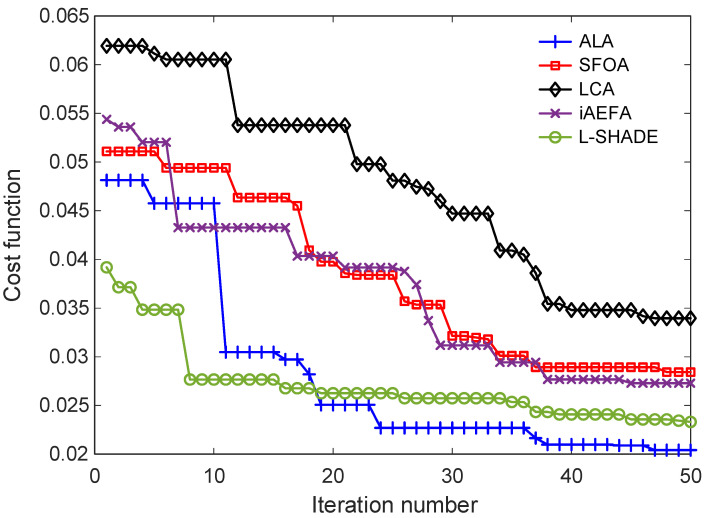
Cost function value minimization with respect to iterations.

The performance of the proposed ALA-based tanh-PID controller and its counterparts, optimized using various metaheuristic algorithms, was first evaluated based on their ability to minimize the defined cost function. This cost function, which integrates time-domain performance indices such as rise time, settling time, overshoot, and steady-state error, serves as the primary criterion for controller optimization. Figure 8 illustrates the convergence behavior of the cost function for all considered algorithms across iterations. As shown, the ALA-based approach consistently achieves a lower cost value and converges more quickly than the other methods. This indicates both its superior exploitation capability and stability during the optimization process. While algorithms such as L-SHADE and iAEFA also reach reasonably low cost values, their convergence curves exhibit longer plateaus or oscillatory behavior, reflecting either slower convergence rates or premature stagnation. The SFOA and LCA-based methods, although capable of minimizing the cost to a certain extent, converge to relatively higher final values, suggesting suboptimal performance in the context of this application.

It should be noted that the noticeable differences in the initial cost function values among the algorithms in Figure 8 primarily originate from their stochastic population initialization and distinct exploration mechanisms. Each optimizer begins the search with randomly distributed candidate solutions within the predefined bounds, and the quality of these initial populations may vary significantly. Furthermore, the employed metaheuristics utilize different early-stage search dynamics (e.g., Brownian-based migration in ALA, differential evolution operators in L-SHADE, and attraction–repulsion mechanisms in iAEFA), which influence how quickly promising regions of the search space are identified. As a result, disparities in the early iterations are expected and do not necessarily reflect the final optimization capability of the algorithms. The later-stage convergence behavior provides a more reliable indicator of overall optimizer performance.

The specific values of the optimized controller parameters resulting from each algorithm are reported in Table 5. Each controller is characterized by eight parameters: the three traditional gains of the tanh-PID controller $\;(K_{p},K_{i},K_{d})$, the filter coefficient, and additional nonlinear and structural parameters that influence control behavior, including shaping factors and tunable nonlinear weights. Notably, the ALA-based tanh-PID controller identifies a unique set of parameters that balance aggressive control gains for instance, $K_{d}\;$ = −8.7905, with higher shaping terms for instance, 2.5264 and 2.8901 and a tuned nonlinearity factor (0.1183) that enables smoother transitions and better noise rejection.

The SFOA-based controller, in contrast, shows an extremely large negative derivative gain ($K_{d}$ = −30.7430), which could lead to excessive sensitivity to noise and instability. Similarly, LCA and iAEFA provide solutions with comparatively moderate gain values but diverging behaviors in other structural parameters. For example, the shaping factor and filter-related parameters in LCA are noticeably smaller than those of ALA, potentially indicating reduced damping and slower settling. L-SHADE’s optimization resulted in relatively balanced parameters but at the cost of higher computational complexity and slower convergence. Overall, the ALA-based approach yields a set of parameters that not only lead to faster convergence and lower cost but also reflect a control structure tuned for precision, stability, and responsiveness. This optimized configuration plays a foundational role in the superior transient and steady-state performance metrics discussed in the following sections.

### 6.5. Transient Performance Analysis

**Figure 9:**
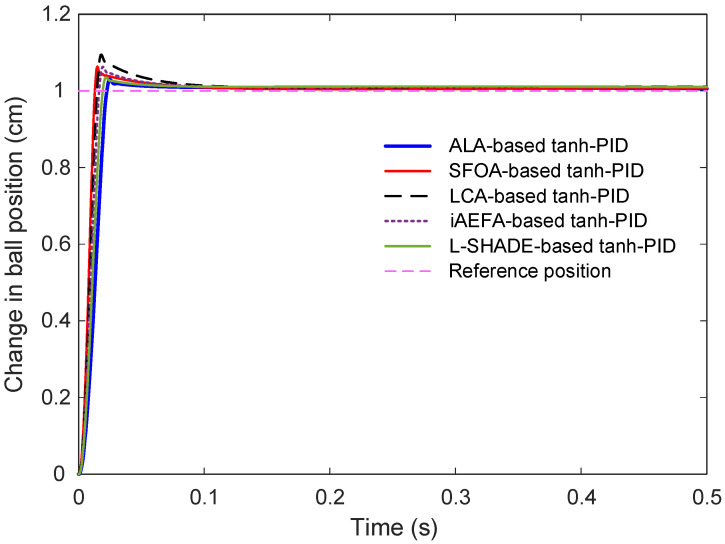
Comparative responses of tanh-PID controllers optimized by different algorithms.

**Figure 10:**
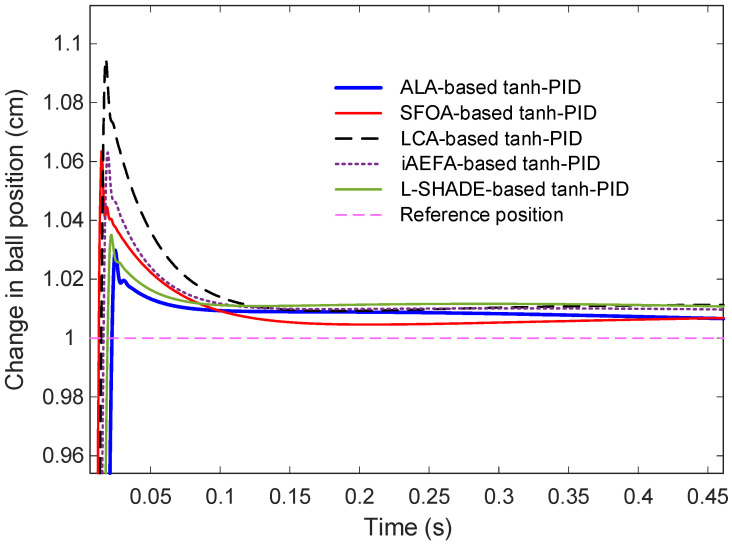
Zoomed view of comparative step response showing the change in ball position.

This section presents a detailed comparison of the transient response characteristics of the proposed ALA-based tanh-PID controller against several recent variants of tanh-PID controllers that were optimized using different metaheuristic algorithms. Specifically, the controllers based on SFOA, LCA, iAEFA, and L-SHADE were evaluated. All controllers Figure 9 displays the comparative step response of the different control methods. It is evident that all controllers successfully guide the system toward the desired reference. However, the trajectories reveal notable differences in response speed, peak amplitude, and damping behavior. To better observe the initial dynamic behavior and slight variations in settling patterns, Figure 10 provides a zoomed-in view of the same responses. From this magnified perspective, the superior behavior of the ALA-based controller becomes clearer, particularly in its ability to minimize overshoot and reach steady-state rapidly were subjected to a unit step input to assess their ability to regulate the ball position effectively and rapidly.

Quantitative performance metrics extracted from the step responses are summarized in Table 6. These include rise time, settling time, peak value, overshoot, and steady-state error. The ALA-based tanh-PID controller achieves the most balanced and consistent performance across all metrics. With a rise time of 0.0144 s and a settling time of only 0.0275 s, it offers a rapid yet stable transition to the desired state. Although SFOA- and LCA-based controllers exhibit slightly faster rise times (0.0081 s and 0.0093 s, respectively), their responses are accompanied by significantly higher overshoot levels 6.35% for SFOA and 9.49% for LCA compared to just 2.98% for the ALA-based controller.

In terms of peak output, the ALA-based controller maintains the closest value to the reference (1.0298 cm), indicating precise amplitude control. Other methods produce higher peak deviations, with LCA-based tanh-PID reaching 1.0949 cm, suggesting a less damped response. Steady-state error is another important performance criterion. Here again, the ALA-based controller excels, achieving a negligible error of 2.6898 × 10^−5^, which is lower than those of all other methods, including the L-SHADE-based controller (9.1299 × 10^−5^) and iAEFA-based controller (3.8023 × 10^−4^). These results highlight the effectiveness of the proposed controller in ensuring fast rise, rapid stabilization, and minimal overshoot while preserving excellent steady-state accuracy. The use of the ALA allows for dynamic adjustment of controller gains in real time, which enhances robustness and responsiveness. Moreover, the tanh nonlinearity contributes to smooth control action and helps limit overshoot by gradually saturating control effort in critical transitions. Generally, while some controllers show isolated advantages in individual metrics, only the ALA-based tanh-PID achieves consistently high performance across all key transient parameters. This makes it a compelling choice for systems requiring both speed and stability, especially in applications where overshoot and precision are critical.

### 6.6. Comparison with Reported Ideal PID-Based Control Methods

To further validate the performance of the proposed ALA-based tanh-PID controller, it is compared against several benchmark ideal PID controllers that were previously tuned using metaheuristic algorithms, including SCA-based PID [48], WDO-based PID [48], WOA-based PID [29] and SA-based PID [29]. These traditional PID controllers follow the classical structure involving only proportional, integral, and derivative terms, but rely entirely on offline optimization for tuning their gains.

(13) $${PID}_{ideal}\left(s\right)=K_{p}+\frac{K_{i}}{s}+K_{d}s$$

#### 6.6.1. Controller Parameters

The optimized gain values obtained for each method are presented in Table 7. A wide variation in the controller parameters is observed, which reflects the influence of each algorithm’s exploration–exploitation balance. For instance, the WOA-based PID shows relatively moderate gain values, whereas the SCA- based PID and WDO-based PID methods yield considerably more negative derivative gains, suggesting more aggressive damping behavior.

#### 6.6.2. Step Response Analysis

**Figure 11:**
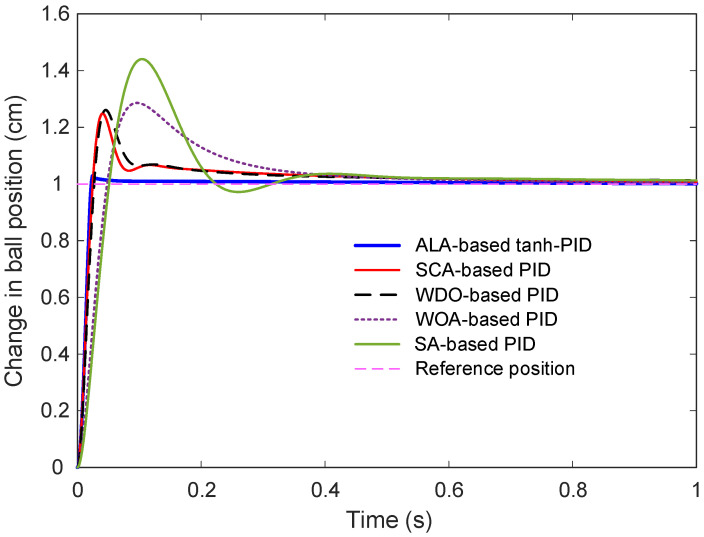
Comparative step response with respect to PID based approaches.

The comparative dynamic behavior of these controllers under a unit step input is illustrated in Figure 11. It is immediately evident that the proposed ALA-based tanh-PID achieves a superior dynamic response in terms of both speed and smoothness. The response curve is sharply rising and stabilizes quickly with minimal overshoot. In contrast, the conventional PID methods exhibit significantly delayed settling, larger peak deviations, and excessive overshoots, especially in the case of the SA-based PID, which shows a visibly oscillatory response before reaching stability.

#### 6.6.3. Performance Metrics

The quantitative time-domain metrics are listed in Table 8. The ALA-based tanh-PID controller outperforms all other methods in every metric. It demonstrates the fastest rise time of 0.0144 s, whereas the next best, the SCA-based PID, takes 0.0168 s, and the slowest method, SA-based PID, takes 0.0358 s. This advantage in responsiveness is even more striking in the settling time, where the proposed controller stabilizes in just 0.0275 s, compared to over 0.5 s for all PID variants. In terms of peak value and overshoot, the proposed controller again maintains better performance, peaking at 1.0298 cm with only 2.98% overshoot. All other methods exhibit far greater peak values and overshoots, most notably; the SA-based PID reaches 1.4407 cm and a staggering 44.07% overshoot, which is unsuitable for applications requiring stability and precision. The steady-state error also demonstrates the accuracy of the proposed controller. It achieves an error of only 2.6898 × 10^−5^, significantly smaller than those of all other methods, with WDO-based PID and SA-based PID reaching errors of 0.0338 and 0.0076, respectively. Although the WOA-based PID shows a numerically small error, its high overshoot and longer settling time diminishes its practical effectiveness.

The improved performance of the ALA-based tanh-PID controller can be attributed to its nonlinear design. Unlike conventional PID methods, which rely on static gain values optimized offline, the proposed controller incorporates an ALA that continuously tunes the control action in response to real-time feedback. Additionally, the inclusion of the tanh nonlinearity introduces smooth saturation behavior, which limits excessive control effort and prevents instability, particularly during abrupt transitions. Generally, while the compared ideal PID controllers demonstrate acceptable performance within the limitations of their fixed structure and tuning approach, they are outclassed by the ALA-based tanh-PID in every respect. The proposed method offers faster response, more accurate tracking, minimal overshoot, and negligible steady-state error, all while maintaining a simpler control architecture. These results confirm the potential of the ALA-based tanh-PID controller as a high-performance and practical solution for real-world control applications.

### 6.7. Comparison with Reported FOPID-Based Control Methods

To assess the effectiveness of the proposed ALA-based tanh-PID controller, it is compared with several prominent fractional-order PID (FOPID) control methods reported in [30], namely: ObAEF-based FOPID, AEF-based FOPID, ASO-based FOPID, and ABC-based FOPID. Each of these approaches employs a different metaheuristic optimization algorithm to tune the five control parameters of a FOPID controller, which includes not only the traditional proportional $K_{p}$, integral $K_{i}$, and derivative $K_{d}$ gains but also the fractional orders of integration λ and differentiation μ.

(14) $$FOPID\left(s\right)=K_{p}+\frac{K_{i}}{s^{\lambda }}+K_{d}s^{\mu }$$

#### 6.7.1. Controller Parameters

Table 9 presents the optimized parameters for each method. A clear variation in parameter tuning is observed, with some algorithms favoring larger gains for instance ABC and ObAEF with highly negative $K_{p}$ and others adjusting the fractional powers differently. The ObAEF and ABC approaches, for example, set higher fractional derivative orders μ>0.1 potentially indicating an attempt to increase system responsiveness, albeit at the cost of noise sensitivity and potential overshoot.

#### 6.7.2. Step Response Analysis

**Figure 12:**
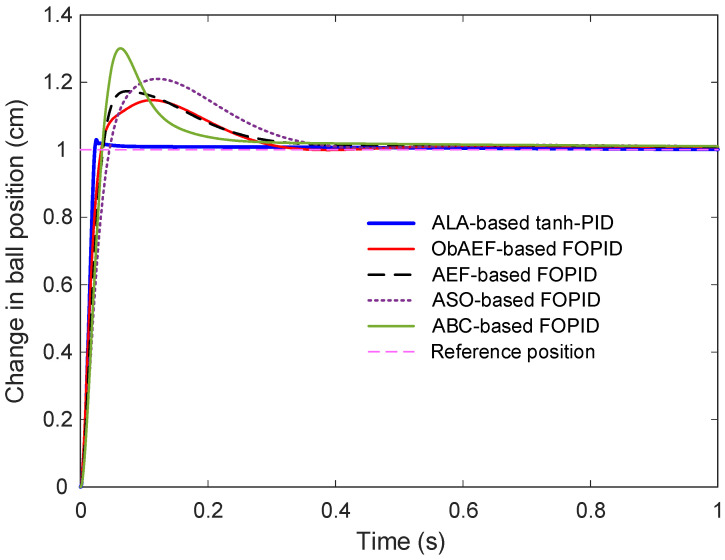
Comparative step response with respect to FOPID based approaches.

The step response comparison, shown in Figure 12, further clarifies the impact of these tunings. The proposed ALA-based tanh-PID controller achieves significantly better transient characteristics. Its response is sharply rising, smoothly converging, and free of the excessive overshoot that is clearly evident in the curves of the FOPID-based controllers. Specifically, all the FOPID methods demonstrate sluggish convergence and varying degrees of overshoot, with ABC-based FOPID showing the most pronounced overshoot and peak value.

#### 6.7.3. Performance Metrics

Quantitative evaluation of these dynamic responses is summarized in Table 10, which reports the rise time, settling time, peak value, overshoot percentage, and steady-state error for each control method. The proposed controller exhibits the fastest rise time (0.0144 s), outperforming all FOPID variants, whose rise times range from 0.0233 s to 0.0328 s. Similarly, the ALA-based tanh-PID achieves a remarkably short settling time of 0.0275 s, while the best performing FOPID method, ObAEF-based, requires more than 0.28 s, an order of magnitude slower. This highlights the significant improvement in response speed offered by the proposed method. The peak output and overshoot values further reinforce this performance gap. While the ALA-based controller peaks at 1.0298 cm with only 2.98% overshoot, all FOPID-based methods exceed 1.14 cm in peak value and 14% in overshoot. Notably, the ABC-based FOPID reaches a peak of 1.2783 cm and an overshoot of 27.82%, which may be unsuitable for applications demanding tight output regulation. In terms of accuracy, the steady-state error of the proposed controller is extremely low, just 2.6898 × 10^−5^ whereas the FOPID-based approaches exhibit significantly larger errors, ranging from 0.0285% to as high as 0.2167%. This again underscores the precise regulation capability of the ALA-based tanh-PID structure. Although FOPID controllers offer a flexible framework by generalizing PID dynamics to non-integer orders, their performance is highly sensitive to parameter tuning. Moreover, their reliance on fixed offline optimization limits adaptability. In contrast, the ALA-based tanh-PID controller not only simplifies the control design by avoiding fractional dynamics but also integrates an adaptive learning mechanism and a smooth nonlinearity that together result in superior responsiveness, minimal overshoot, and excellent steady-state performance. These features make the proposed approach a compelling and practical alternative to more complex FOPID schemes, particularly in real-time or precision-critical applications.

### 6.8. Comparison with Reported Real PID Plus Second Order Derivative (RPIDD^2^)-Based Control Methods

In this section, the performance of the proposed ALA-based tanh-PID controller is compared with four well-established RPIDD^2^-based control strategies: MRFO-based RPIDD^2^, AOA-based RPIDD^2^, LFD-based RPIDD^2^, and AEO-based RPIDD^2^, all of which are reported in [33]. These methods leverage nature-inspired optimizers to fine-tune the gains of a sophisticated control structure the RPIDD^2^ controller defined as follows:

(15) $$RPIDD^{2}(s)=K_{p}+\frac{K_{i}}{s}+K_{d1}\left(\frac{N_{1}s}{s+N_{1}}\right)+K_{d2}{\left(\frac{N_{2}s}{s+N_{2}}\right)}^{2}$$

This formulation extends the conventional PID structure by incorporating two derivative terms: a first-order derivative with a low-pass filter and a second-order filtered derivative component.

Where $K_{p}$, $K_{i}$, $K_{d1}$, $K_{d2}$, $N_{1}$ and $N_{2}$ are the proportional gain, the integral gain, the first and second derivative gains, filter coefficients used to attenuate high-frequency noise, respectively.

#### 6.8.1. Controller Parameters

Table 11 presents the tuned parameters obtained from each RPIDD^2^-based method. The diversity of the values reflects the different convergence behaviors of each metaheuristic algorithm. Notably, the MRFO approach results in very high values for $K_{d2}$ or the associated filter parameter, possibly indicating a strong emphasis on second-order derivative control. On the other hand, the AOA and LFD-based controllers adopt relatively milder values across parameters, aiming for a balanced control response.

#### 6.8.2. Step Response Analysis

**Figure 13:**
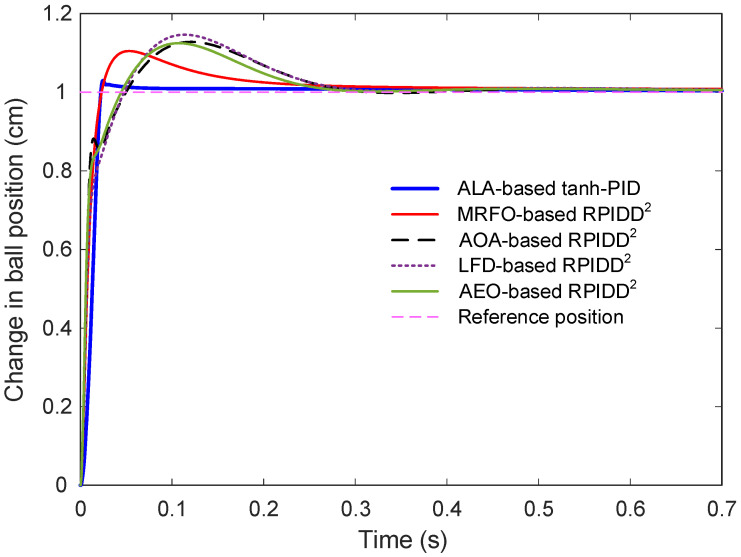
Comparative step response with respect to RPIDD^2^ based approaches.

The time-domain behavior of all controllers under a unit step input is shown in Figure 13. Visually, the ALA-based tanh-PID controller demonstrates superior performance in both rise and settling time. While RPIDD^2^-based methods exhibit visible overshoot and longer convergence times, the proposed controller stabilizes quickly with minimal transient fluctuation. The MRFO- and AEO-based RPIDD^2^ controllers, in particular, show notable overshoot and sluggish decay, indicating less favorable dynamic behavior.

#### 6.8.3. Performance Metrics

Table 12 provides a quantitative comparison of the time-domain performance metrics for all control methods under study, including rise time, settling time, peak response, overshoot, and steady-state error. The results clearly indicate that the proposed ALA-based tanh-PID controller consistently outperforms the RPIDD^2^-based approaches across all evaluated criteria. It achieves the fastest rise time of 0.0144 s, demonstrating a highly responsive behavior to input changes. Moreover, its settling time is remarkably short, stabilizing the output in just 0.0275 s, substantially quicker than the RPIDD^2^-based methods, which all require more than 0.23 s to settle. In terms of peak output, the ALA-based tanh-PID reaches 1.0298 cm, making it the closest to the ideal value of 1 cm. Other controllers exhibit peak responses exceeding 1.1 cm, which implies more pronounced overshoot and less accurate transient performance. This is further evidenced by the overshoot percentage: the proposed controller exhibits a modest 2.98% overshoot, whereas all RPIDD^2^ variants show considerably higher values, often surpassing 10%. Finally, the steady-state error attained by the ALA-based controller is exceptionally low just 2.6898 × 10^−5^, which is significantly smaller than that achieved by any of the benchmark methods, reflecting a substantial improvement in control accuracy and precision.

The superior performance of the proposed controller can be attributed to its underlying design philosophy, which combines an ALA for dynamic, real-time parameter tuning with a hyperbolic tangent based nonlinear preprocessing stage. The ALA enhances adaptability and robustness under varying operating conditions, while the smooth nonlinearity introduced by the tanh function naturally bounds and scales error and derivative signals, preventing control saturation and reducing overshoot. This combination allows the tanh-PID to effectively handle the inherent instability and strong nonlinearity of the magnetic ball suspension system. In contrast, FOPID and RPIDD^2^ controllers, despite their more complex fractional-order or multi-derivative structures, rely on fixed or offline-optimized parameters, making them less effective in coping with time-varying dynamics and disturbances. Simulation results confirm that the proposed approach achieves faster settling, lower overshoot, and better disturbance rejection, offering a more robust and stable control solution for nonlinear and unstable maglev systems.

The improved transient behavior of the proposed controller can be interpreted from both control and optimization perspectives. From the control side, the embedded hyperbolic tangent mapping acts as a smooth nonlinear preprocessing stage that effectively compresses large error magnitudes during the initial transient. Unlike hard saturation or purely linear amplification, the tanh function provides a continuous soft-limiting effect that reduces the effective loop gain when the error is large, thereby mitigating excessive control effort and suppressing overshoot. As the error approaches zero, the tanh function operates in its quasi-linear region, preserving fine regulation accuracy and steady-state precision. This dual-region behavior enables fast yet well-damped responses, which is particularly beneficial for the inherently unstable magnetic ball suspension dynamics.

From the optimization perspective, the superior tuning performance of ALA is attributed to the cooperative action of its four behavioral strategies. The long-distance migration phase promotes wide-range global exploration through stochastic Brownian perturbations, reducing the risk of premature convergence. The digging phase introduces directed local search around promising regions, improving solution refinement. During the foraging stage, Lévy-flight-based movements enable adaptive step sizes that enhance the probability of escaping shallow local minima. Finally, the evasion mechanism provides diversity reinforcement and prevents population stagnation in later iterations. The dynamic energy factor governing the transition among these phases allows ALA to maintain an effective exploration–exploitation balance throughout the search process. This balanced search behavior is particularly advantageous for the highly coupled and multimodal parameter landscape of the tanh-PID controller. Collectively, the bounded nonlinear signal shaping and the adaptive multi-phase search capability of ALA form a complementary design that explains the observed improvements in overshoot reduction, settling speed, and overall closed-loop smoothness.

## 7. Conclusions

In this study, we proposed a novel control approach for magnetic ball suspension systems by integrating a hyperbolic tangent–based PID controller with the artificial lemming algorithm for parameter tuning. The controller design employs a smooth nonlinear preprocessing stage that reshapes the error signals and an adaptive cost function that balances overshoot suppression with tracking accuracy. Extensive simulation results demonstrate that the ALA-tuned tanh-PID controller consistently outperforms classical PID, FOPID, and RPIDD^2^ controllers across all major performance indices, including rise time (0.0144 s), settling time (0.0275 s), peak value (1.0298 cm), overshoot (2.98%), and steady-state error (2.68 × 10^−5^). Despite these promising outcomes, the study has several inherent limitations that must be acknowledged. The current analysis is based on a linearized representation of the MBS system, which may not fully capture large-signal nonlinear dynamics, actuator constraints, or unmodeled uncertainties under extreme operating conditions. Furthermore, real-time hardware validation has not been conducted, and all evaluations have been carried out in a simulation environment. Accordingly, practical deployment aspects (such as execution timing, sensor noise, and actuator saturation) have not been explicitly examined. It is therefore emphasized that the reported results demonstrate benchmark-level stabilization and performance improvement for the standard linearized MBS model, rather than global guarantees for the full nonlinear plant. At the same time, the proposed scheme retains the fundamental simplicity of a classical PID controller. During closed-loop operation, the control law involves only standard PID computations augmented by smooth hyperbolic tangent mappings and a filtered derivative term, resulting in a computational burden comparable to that of a conventional PID controller. The ALA operates solely in an offline, supervisory tuning phase and does not impose any overhead on the real-time control loop. These characteristics make the approach inherently suitable for implementation on low-cost embedded platforms should real-time deployment be pursued. The proposed method has significant potential for broader industrial and engineering applications involving nonlinear, open-loop unstable systems, such as magnetic levitation transport, biomedical levitation platforms, and precision positioning stages. From a systems-design perspective, the availability of a supervisory optimization framework reduces the need for repeated manual retuning and can support more autonomous and cost-effective control solutions.

Future work may extend the controller to multi-input multi-output (MIMO) configurations, incorporate real-time hardware-in-the-loop experimentation, and investigate hybrid schemes that combine ALA with learning-based components for improved generalization in highly dynamic environments. In addition, integrating sensor fusion and fault-diagnosis layers could further enhance resilience and reliability for deployment in safety-critical applications.

## Figures and Tables

**Table 1:** Parameters of the examined system.

Parameter	Value	Unit
R (winding resistance)	1	Ω
L (winding inductance)	0.01	H
k (electromagnet coefficient)	1	kg × m^2^/A^2^ × s^2^
g (gravitational acceleration)	9.81	m/s^2^
M (mass of the ball)	1	kg
$y_{0}$ (equilibrium point)	0.5	m

**Table 2:** The parameter boundaries adopted for this study.

Constraint	$K_{p}$	$K_{i}$	$K_{d}$	N	$\tau_{1}$	$G_{1}$	$\tau_{2}$	$G_{2}$
Lower	−100	−100	−100	1	0.1	0.1	0.1	0.1
Upper	−0.1	−0.1	−0.1	1000	3	3	3	3

**Table 3:** Parameter settings of ALA, SFOA, LCA, iAEFA and L-SHADE.

Algorithm	Reference	Parameter	Value
ALA	[8]	Switching probability Prob	0.3
SFOA	[21]	Algorithmic parameter $G_{P}$	0.5
LCA	[22]	Tumor constant f	1
iAEFA	[23]	Initial Coulomb’s constant $K_{0}$	500
		Control parameter α	40
L-SHADE	[47]	Crossover rate $M_{CR}$	0.5
		Scaling factor $M_{F}$	0.5

**Table 4:** Comparative numerical values showing the statistical performance.

Algorithm	Minimum	Maximum	Average	Standard Deviation	Rank
ALA	**2.0414 × 10^−2^**	**2.4413 × 10^−2^**	**2.2148 × 10^−2^**	**1.2142 × 10^−3^**	**1**
SFOA	2.8424 × 10^−2^	3.4572 × 10^−2^	3.0730 × 10^−2^	1.8099 × 10^−3^	4
LCA	3.3960 × 10^−2^	4.0321 × 10^−2^	3.7037 × 10^−2^	1.8563 × 10^−3^	5
iAEFA	2.7285 × 10^−2^	3.3048 × 10^−2^	2.9668 × 10^−2^	1.5715 × 10^−3^	3
L-SHADE	2.3310 × 10^−2^	2.8706 × 10^−2^	2.5214 × 10^−2^	1.4255 × 10^−3^	2

**Table 5:** Optimized tanh-PID controller parameters via ALA, SFOA, LCA, iAEFA, L-SHADE.

Algorithm	$K_{p}$	$K_{i}$	$K_{d}$	N	$\tau_{1}$	$G_{1}$	$\tau_{2}$	$G_{2}$
ALA	−39.4756	−82.0490	−8.7905	805.2558	2.8901	2.5264	0.1183	1.4294
SFOA	−35.6523	−82.5568	−30.7430	892.7579	1.9395	2.0489	0.1607	1.1084
LCA	−50.7021	−74.9492	−20.5509	940.0705	1.3886	2.0974	0.1244	0.7728
iAEFA	−53.4635	−61.2643	−14.3121	904.6210	1.5021	2.5728	0.1023	1.0386
L-SHADE	−46.5113	−81.6004	−12.8757	988.8356	1.8015	2.1368	0.1047	0.9373

**Table 6:** Comparative numerical values showing the time domain performance metrics.

Control Method	Rise Time (s)	Settling Time (s)	Peak (cm)	Overshoot (%)	Steady-State Error (%)
ALA-based tanh-PID	0.0144	**0.0275**	**1.0298**	**2.9818**	**2.6898 × 10^−5^**
SFOA-based tanh-PID	**0.0081**	0.0559	1.0635	6.3466	0.0760
LCA-based tanh-PID	0.0093	0.0777	1.0949	9.4880	0.0059
iAEFA-based tanh-PID	0.0104	0.0591	1.0630	6.3018	3.8023 × 10^−4^
L-SHADE-based tanh-PID	0.0122	0.0389	1.0351	3.5072	9.1299 × 10^−5^

**Table 7:** Reported parameters for PID based approaches.

Algorithm	$K_{p}$	$K_{i}$	$K_{d}$
SCA	−73.0739	−98.5832	−9.8736
WDO	−81.1284	−65.3098	−8.4581
WOA	−39.9633	−75.4406	−3.8101
SA	−52.9824	−60.5218	−2.8532

**Table 8:** Comparative time domain performance metrics with respect to PID based approaches.

Control Method	Rise Time (s)	Settling Time (s)	Peak (cm)	Overshoot (%)	Steady-State Error (%)
ALA-based tanh-PID	**0.0144**	**0.0275**	**1.0298**	**2.9818**	2.6898 × 10^−5^
SCA-based PID	0.0168	0.5506	1.2487	24.8724	4.5351 × 10^−4^
WDO-based PID	0.0185	0.5735	1.2611	26.1061	0.0338
WOA-based PID	0.0334	0.5089	1.2861	28.6102	**1.2322 × 10^−5^**
SA-based PID	0.0358	0.6031	1.4407	44.0653	0.0076

**Table 9:** Reported parameters for FOPID based approaches.

Algorithm	$K_{p}$	$K_{i}$	$K_{d}$	λ	μ
ObAEF	−82.0892	−95.6888	−3.2004	0.7769	1.2403
AEF	−72.8609	−81.0298	−3.6139	0.9812	1.1496
ASO	−49.5017	−69.9558	−2.7166	0.8845	1.1598
ABC	−83.7975	−77.3375	−5.0983	0.9905	1.0293

**Table 10:** Comparative time domain performance metrics with respect to FOPID based approaches.

Control Method	Rise Time (s)	Settling Time (s)	Peak (cm)	Overshoot (%)	Steady-State Error (%)
ALA-based tanh-PID	**0.0144**	**0.0275**	**1.0298**	**2.9818**	**2.6898 × 10^−5^**
ObAEF based FOPID	0.0233	0.2873	1.1487	14.8608	0.2167
AEF-based FOPID	0.0257	0.3300	1.1718	17.1741	0.0285
ASO-based FOPID	0.0328	0.3562	1.2113	21.1222	0.1326
ABC-based FOPID	0.0246	0.3448	1.2783	27.8227	0.0316

**Table 11:** Reported parameters for RPIDD^2^ based approaches.

Algorithm	$K_{p}$	$K_{i}$	$K_{d1}$	$N_{1}$	$K_{d2}$	$N_{2}$
MRFO	−145.8225	−139.5592	−11.4316	595.5822	−0.1140	966.3598
AOA	−107.4512	−174.0175	−6.7517	655.5316	−0.2114	560.5807
LFD	−94.2834	−146.7221	−6.2381	883.2237	−0.1526	746.8765
AEO	−119.2157	−184.1486	−7.4718	958.5826	−0.1909	770.8188

**Table 12:** Comparative time domain performance metrics with respect to RPIDD^2^ based approaches.

Control Method	Rise Time (s)	Settling Time (s)	Peak (cm)	Overshoot (%)	Steady-State Error (%)
ALA-based tanh-PID	**0.0144**	**0.0275**	**1.0298**	**2.9818**	**2.6898 × 10^−5^**
MRFO-based RPIDD^2^	0.0145	0.2329	1.1048	10.4824	0.0086
AOA-based RPIDD^2^	0.0274	0.2586	1.1281	12.8087	2.0170 × 10^−4^
LFD-based RPIDD^2^	0.0288	0.2602	1.1465	14.6456	3.1843 × 10^−4^
AEO-based RPIDD^2^	0.0247	0.2389	1.1248	12.4800	3.0027 × 10^−4^

## Data Availability

All produced data are available within the manuscript.
